# Three-dimensional printing and virtual reconstruction in surgical planning of double-outlet right ventricle repair

**DOI:** 10.1016/j.xjtc.2022.11.005

**Published:** 2022-11-26

**Authors:** Kevin Ponchant, Duy-Anh Nguyen, Milan Prsa, Maurice Beghetti, Tornike Sologashvili, Jean-Paul Vallée

**Affiliations:** a Cardiovascular Radiology Unit, Geneva University Hospitals and University of Geneva, Geneva, Switzerland; b Pediatric Cardiology Unit, Children's University Hospital, Geneva, Switzerland; c Division of Pediatric Cardiology, Woman-Mother-Child Department, Lausanne University Hospital and University of Lausanne, Lausanne, Switzerland; d Centre Universitaire Romand de Cardiologie et Chirurgie Cardiaque Pédiatrique, Geneva University Hospitals/Lausanne University Hospital, Geneva/Lausanne, Switzerland; e Division of Cardiac Surgery, Geneva University Hospitals, Geneva, Switzerland

**Keywords:** double-outlet right ventricle, 3D virtual valvular reconstruction, 3D printed heart model, 3D modality in surgical planning, 3D printing, 3D, 3-dimensional, 3DPHM, 3D-printed heart model, 3DVVR, 3D virtual valvular annulus reconstruction, CTA, computed tomography angiogram, DORV, double-outlet right ventricle, LV, left ventricle, PA, pulmonary artery, PV, pulmonary valve, TGA, transposition of the great arteries, TTE, transthoracic echocardiography, VSD, ventricular septal defect

## Abstract

**Objectives:**

For more than a decade, 3-dimensional (3D) printing has been identified as an innovative tool for the surgical planning of double-outlet right ventricle (DORV). Nevertheless, lack of evidence concerning its benefits encourages us to identify valuable criteria for future prospective trials.

**Methods:**

We conducted a retrospective study involving 10 patients with DORV operated between 2015 and 2019 in our center. During a preoperative multidisciplinary heart team meeting, we harvested surgical decisions following a 3-increment step process: (1) multimodal imaging; (2) 3D virtual valvular reconstruction (3DVVR); and (3) 3D-printed heart model (3DPHM). The primary outcome was the proportion of predicted surgical strategy following each of the 3 steps, compared with the institutional retrospective surgical strategy. The secondary outcome was the change of surgical strategy through 3D modalities compared with multimodal imaging. The incremental benefit of the 3DVVR and 3DPHM over multimodal imaging was then assessed.

**Results:**

The operative strategy was predicted in 5 cases after multimodal imaging, in 9 cases after 3DVVR, and the 10 cases after 3DPHM. Compared with multimodal imaging, 3DVVR modified the strategy for 4 cases. One case was correctly predicted only after 3DPHM inspection.

**Conclusions:**

3DVVR and 3DPHM improved multimodal imaging in the surgical planning of patients with DORV. 3DVVR allowed a better appreciation of the relationships between great vessels, valves, and ventricular septal defects. 3DPHM offers a realistic preoperative view at patient scale and enhances the evaluation of outflow tract obstruction. Our retrospective study demonstrates benefits of preoperative 3D modalities and supports future prospective trials to assess their impact on postoperative outcomes.

**Figure:**
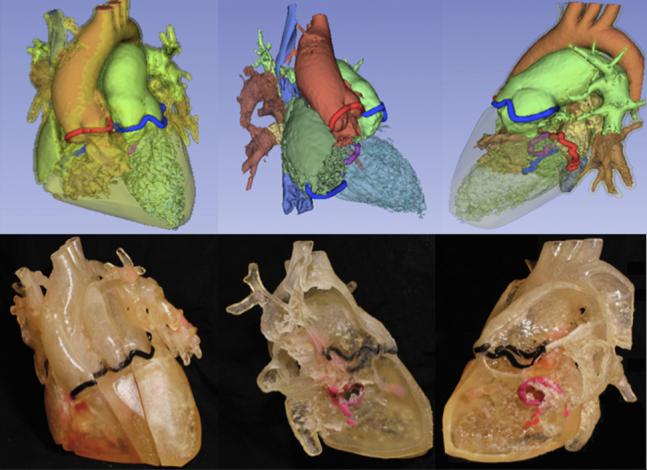
3DVVR and 3DPHM used in the preoperative planning of surgical strategy.

**Central Message**

Both 3DVVR and 3DPHM improved standard multimodal imaging in the definition of surgical strategy of complex DORV repair.

**Perspective**

Future prospective studies would be appropriate to assess the postoperative impact of 3DVVR and 3DPHM in surgical planning on patient short- and long-term outcomes.

Congenital heart disease is a global concern in child and adult health. Without the ability to substantially reduce the prevalence of congenital heart disease, interventions and resources must be invested to improve mortality, operative outcomes, survival, and quality of life.^1^

Double-outlet right ventricle (DORV) is a complex type of ventriculoarterial discordance accounting for 1% to 3% of all congenital heart diseases, with a reported incidence of 3-9/100,000 live births.^2^^,^^3^ As the result of its heterogeneity, each DORV case is unique, making surgical planning of its total repair one of the greatest challenges in the field of congenital heart disease.^4^ Numerous surgical techniques have been validated for the repair of DORV, including intraventricular repair^5^ and arterial switch operation,^6^ the Rastelli procedure,^7^ Réparation à l'Etage Ventriculaire,^8^ the Bex-Nikaidoh procedure,^9^, ^10^, ^11^ and the outflow tract rotation, also known as half-turned truncal switch operation^12^^,^^13^ or en-bloc rotation of the outflow tracts.^14^, ^15^, ^16^, ^17^ The choice between these procedures is often difficult and dictated by the surgeon's preferences as well as the heart anatomy and associated abnormalities. Imaging plays an important role in this assessment.^4^

### Imaging and 3-Dimensional (3D) Printing

Multimodal imaging, including transthoracic echocardiography (TTE), computed tomography angiogram (CTA), and magnetic resonance imaging (MRI), is a key element in surgical planning using both 2-dimensional visualization and well-established 3D-reconstruction techniques. Complex intracardiac anatomy visualization can be improved with new 3D modalities.^18^ In particular, 3D virtual valvular reconstruction (3DVVR) and 3D-printed heart model (3DPHM) have the potential to revolutionize the care of pediatric cardiac patients.^19^ However, their impact on surgical planning is still not well established. Although some studies have tried to demonstrate the utility of 3D printing in surgical planning for patients with DORV,^20^, ^21^, ^22^, ^23^, ^24^, ^25^ Batteux and colleagues^25^ mentioned the lack of evidence for such benefits due to heterogeneity of studied congenital heart disease and suggested that retrospective comparison of 3D models with standard multimodal imaging should be the first step to perform. Therefore, this study aimed to compare retrospectively the added value of 3DVVR and 3DPHM with standard multimodal imaging in the planning of DORV surgical repair.

## Methods

### Study Design

**Figure 1:**
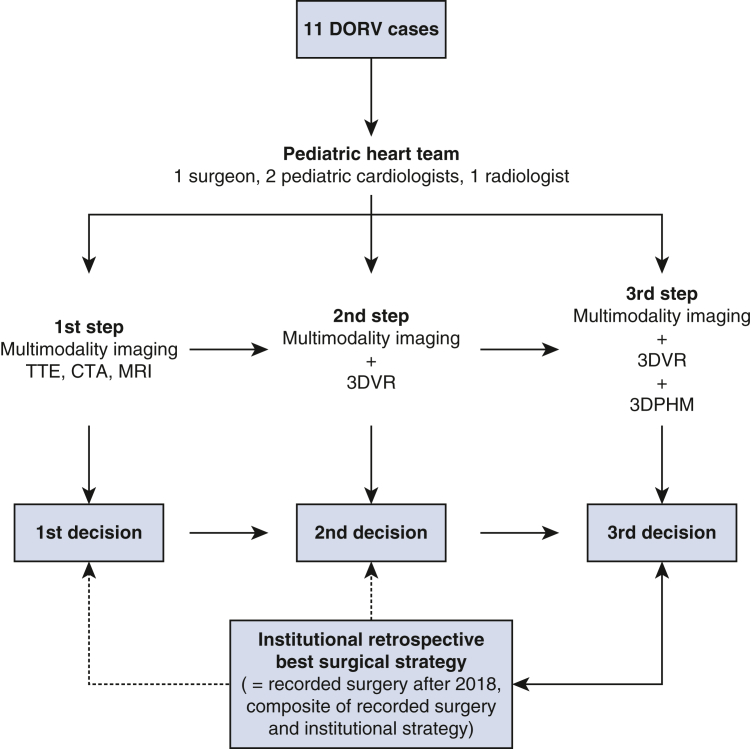
Design of our study. *DORV*, Double-outlet right ventricle; *TTE*, transthoracic echocardiography; *CTA*, computed tomography angiogram; *MRI*, magnetic resonance imaging; *3DVR*, 3-dimensional virtual reconstruction; *3DPHM*, 3-dimensional printed heart model.

We conducted a retrospective study of 10 pediatric patients with DORV who underwent surgical repair by a single surgeon in 2 tertiary hospitals between 2016 and 2019. The study was approved by the institutional review board (authorization 2017-00716, 31.01.2018). The inclusion criteria were patients with DORV transposition of great arteries (TGA) type who could undergo surgical repair and the availability of preoperative echocardiogram as well as a cardiac MRI or CTA. Patient data were deidentified and uploaded to an institutional secured cloud server. Patients were discussed among the members of a multidisciplinary pediatric heart team, which included 2 pediatric cardiologists, 1 cardiac surgeon, and 1 cardiovascular radiologist. The incremental value of 3DVVR and 3DPHM was determined by a 3-step evaluation process (Figure 1).

### Step 1: Multimodality Imaging

**Figure E1:**
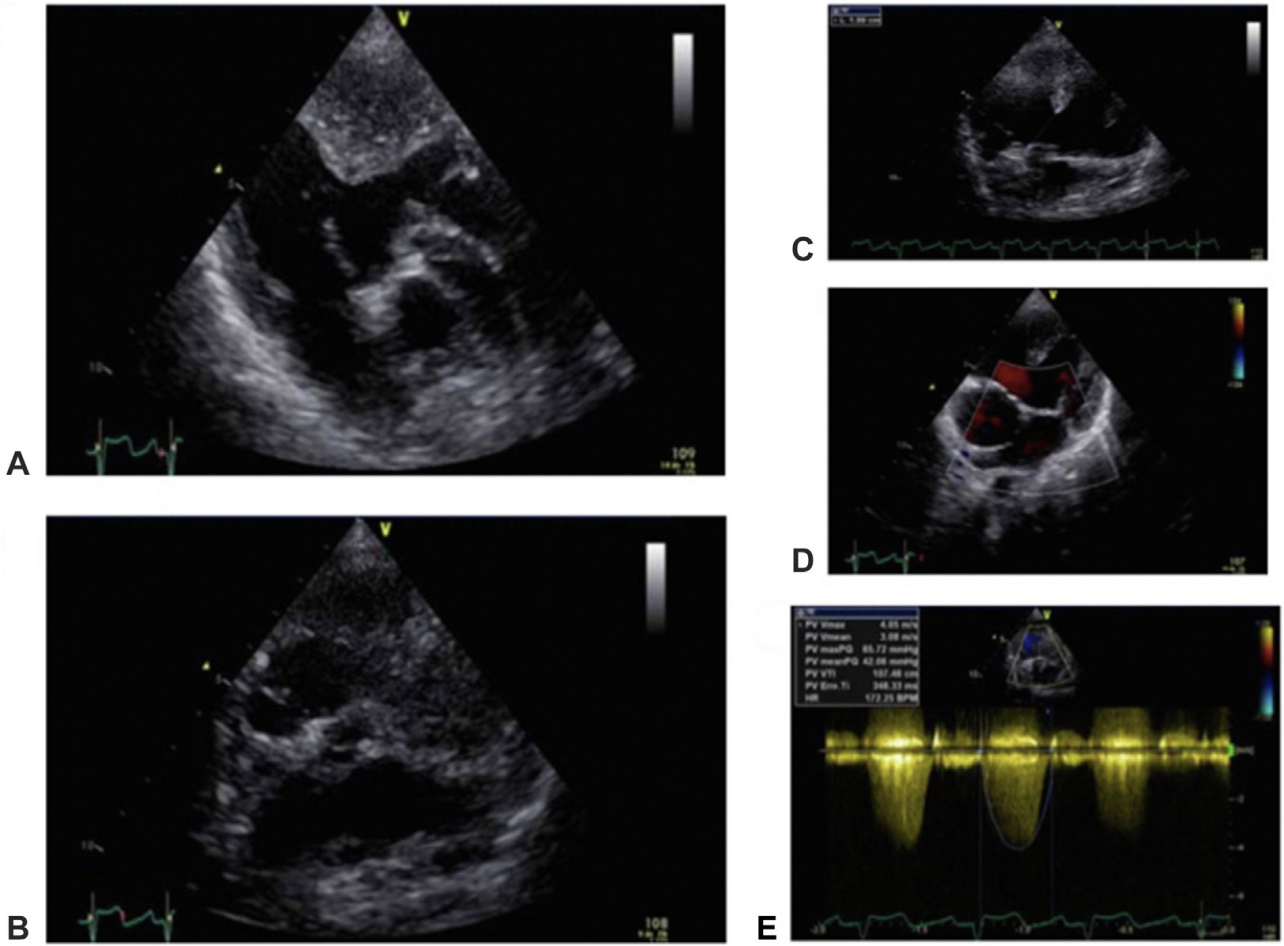
TTE images. A, TGA. B, Size discrepancy between aorta and pulmonary trunk. C, Large subpulmonary VSD. D, VSD with posterior extension. E, Doppler of pulmonary trunk.

**Figure E2:**
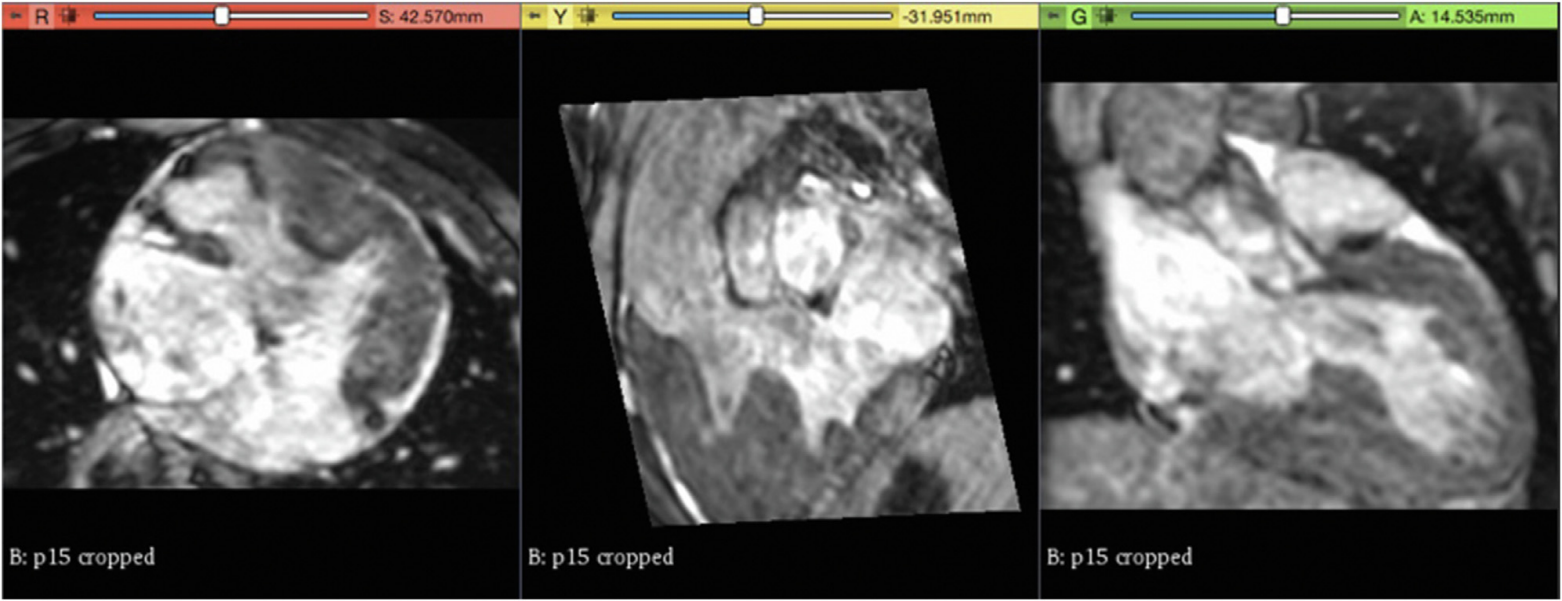
MRI acquisition: TGA with anteroposterior aortopulmonary position, subpulmonary VSD with posterior extension.

All patients had complete TTE, presented by a pediatric cardiologist (Figure E1). Seven patients had MRI, and 3 patients had CTA, including a volume rendering of the blood pool, presented by the cardiovascular radiologist (Figure E2). A first decision about the type of surgical repair was recorded at this point.

### Step 2: 3D Virtual Valvular Annuli Reconstruction

**Figure 2:**
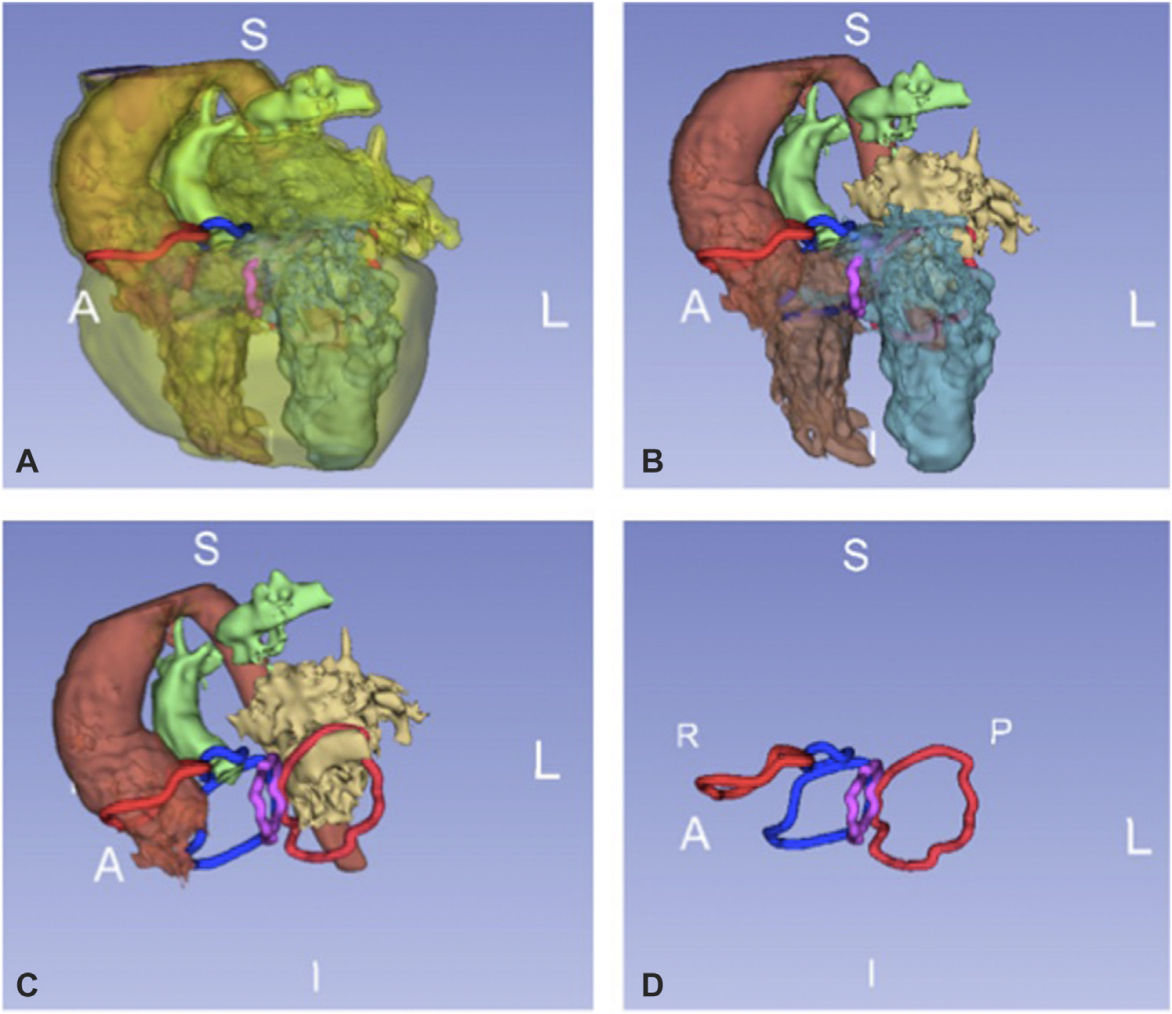
A sequential 3D virtual reconstruction of anatomical segments. (A) Postsegmentation 3D virtual reconstruction. (B) Myocardium is hidden; (C) RA, LV, and RV hidden, to appreciate intervalvular relationship with VSD. Aortic and mitral valves are shown in *red*, pulmonary and tricuspid in *blue*, and VSD in *purple*. (D) Complete valvular visualization with *VSD* in purple. *S*, Superior; *A*, anterior; *L*, left; *R*, right; *P*, posterior; *I*, inferior.

3DVVR was carried out by segmentation of a model from either CTA or MRI scans using the open-source software 3D Slicer.^26^ A semiautomated segmentation was completed by manual correction when needed. All 4 valvular annuli were manually depicted and kept opaque, whereas the blood pool was made semitransparent. Chordal attachment aberrations and straddling were only analyzed on multimodal imaging (TTE, CTA/MRI) and were not represented on 3DVVR nor 3DPHM. Each segment can be selectively faded or hidden, allowing user-defined visualization of the cavities and valves (Figure 2). A second decision about the type of surgical repair was recorded following 3DVVR visualization.

### Step 3: 3DPHM

**Figure E3:**
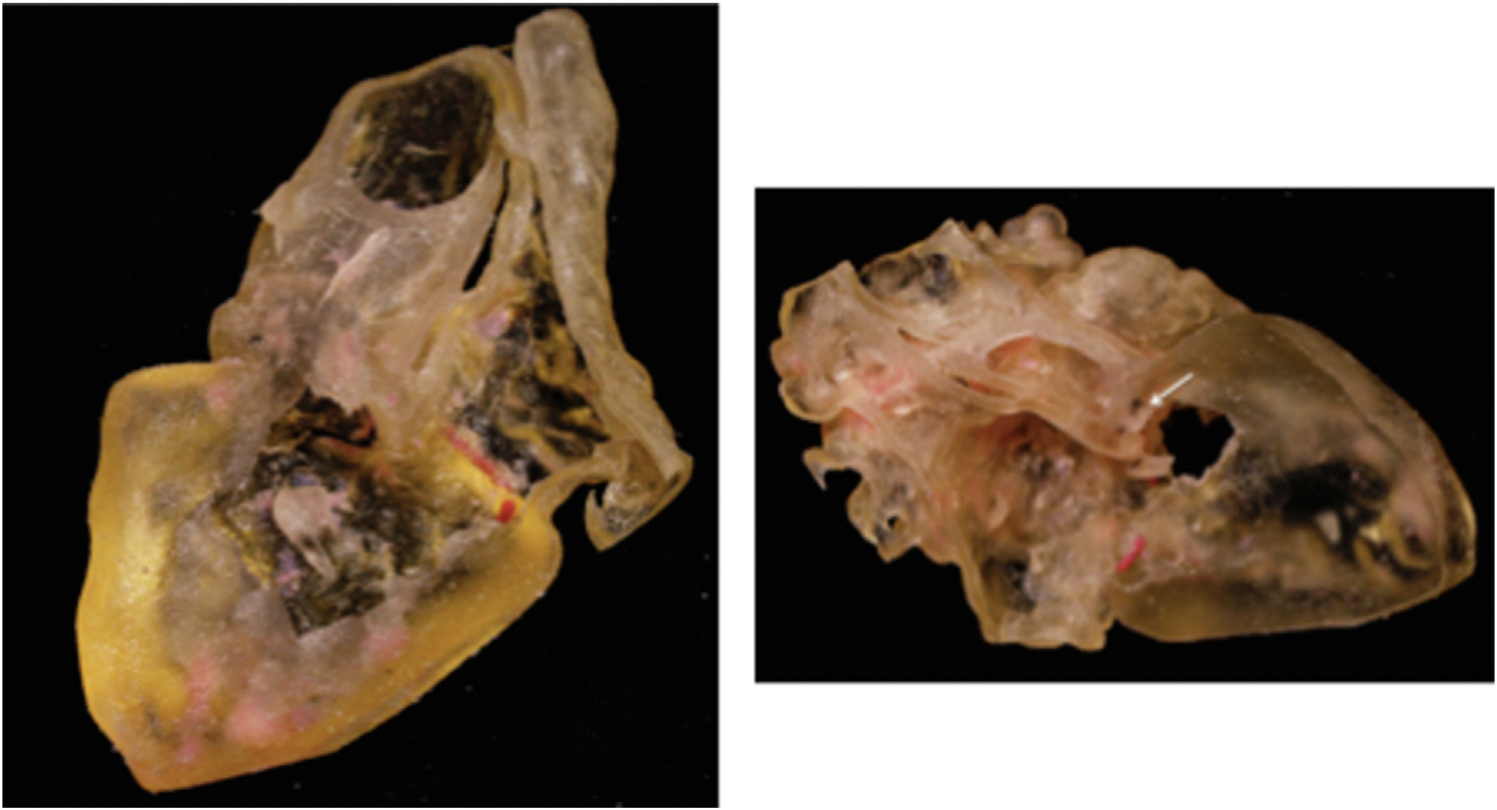
The model allowed a hands-on anatomical analysis with scale respecting dimensions. On the first image (*right*), a pseudo-sagittal cut of the RV can be appreciated. The *black* shows the aortic annulus and *magenta* shows the TV. On the *left*, VSD is subpulmonary, and the pulmonary stenosis is denoted in *black* (*arrow*). *A*, Anterior; *S*, superior; *L*, left.

The process of 3D printing included image segmentation and exporting in standard tessellation language (ie, STL) file format using 3D Slicer, correction of the standard tessellation language model by MeshMixer (Autodesk, Inc) and 3D printing with a Stratasys Objet260 Connex 3 printer (version 29.11.0.19189). The resins used were VeroWhite Plus, VeroBlack, and VeroMagenta for the valves and TangoPlus for the cardiac chambers and vessels. A 1:1 scale 3DPHM (Figure E3) was presented in 3 parasagittal slices allowing complete visualization of cardiac chambers and great vessels. A third and final decision was then recorded.

At the end of the simulation, the previous original heart team decision as well as operative records with perioperative findings and performed surgical procedure were revealed. On the basis of the latter, an institutional retrospective surgical strategy, defined as a composite of the performed procedure and the final simulated heart team decision considering currently available expertise, was finally defined as the gold standard and compared with each of the 3-step decisions.

### Primary and Secondary Outcomes

The primary outcome was the proportion of correctly predicted surgical repair strategies following multimodal imaging, 3DVVR, and 3DPHM. The secondary outcome was the change of surgical strategy between the multimodal imaging step and the two 3D modalities steps (3DVVR and 3DPHM). The incremental benefit of 3DVVR and 3DPHM was then compared separately. We also reported patients' outcomes, such as intensive care unit length of stay, hospitalization length of stay, in-hospital survival, reoperation rate, need of permanent pacemaker, and up-to-follow-up survival.

## Results

Ten patients with DORV who underwent operation in our institution between 2015 and 2019 were included in the study. All had DORV TGA-type with anteroposterior (n = 7) and side-by-side aortopulmonary positions (n = 3). Sex repartition was 6 male and 4 female patients. The mean age of pediatric patients was 4.4 years ± 4.1 years. Mean time between the surgical repair and the study was an average of 19 months (Tables E1 and E2).

Surgical repair included 3 arterial switch operations, 3 Bex-Nikaidoh procedures, 1 intraventricular repair, 1 outflow tract rotation, 1 postponed Bex-Nikaidoh after previous pulmonary artery (PA) banding and atrioseptotomy, and 1 single-ventricle palliation. The retrospective institutional surgical strategies following the 3 steps process resulted in 4 outflow tract rotations, 3 Bex-Nikaidoh procedures, 2 arterial switch operations, and 1 intraventricular repair. There were discrepancies between operative records and simulated heart team final decision for 4 patients resulting from temporal evolution of surgical expertise (for p01, single-ventricle palliation changed to Bex-Nikaidoh; for p03, Bex-Nikaidoh changed to outflow tract rotation; for p05, PA banding and atrioseptotomy before Nikaidoh changed to PA banding and atrioseptotomy before outflow tracts rotation; for p10, arterial switch operation changed to outflow tract rotation).

### Primary Outcome: Predictive Value of Multimodal Imaging and 3D Modalities

**Figure 3:**
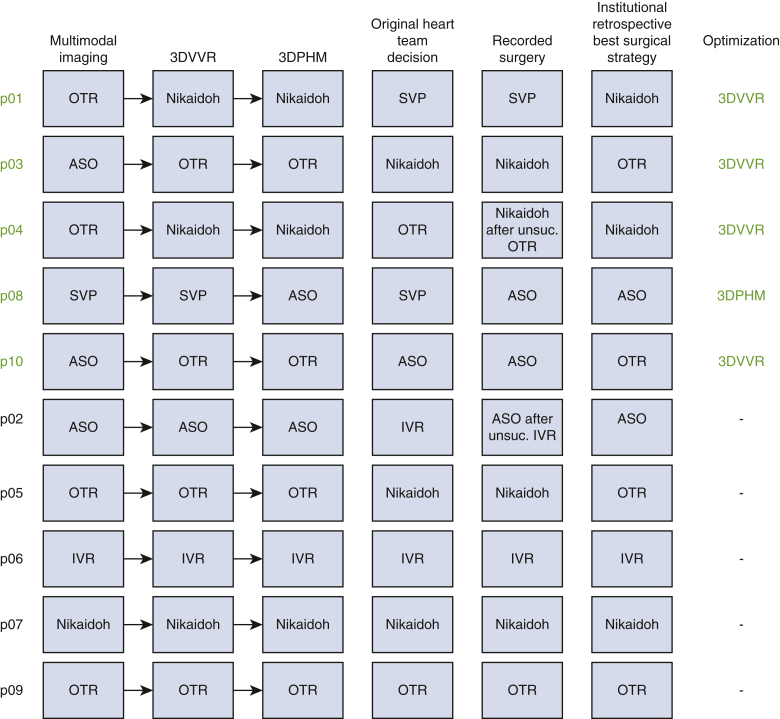
Sequential predicted surgical strategies, compared with original heart team decision, and medical records to define retrospective institutional surgical strategy. *3DVR*, Changes due to 3-dimensional reconstruction; *3DPHM*, change due to printed models; *OTR*, outflow tract rotation; *SVP*, single ventricular palliation; *ASO*, arterial switch operation; *IVR*, intraventricular repair.

Decisions for each case following the 3-step simulation are summarized in Figure 3. According to retrospective institutional surgical strategies, the prediction after multimodal imaging concurred for 5 cases (p02, p05, p06, p07 p09). The evaluation of 3DVVR confirmed the prediction of theses 6 cases and brought 4 additional correct predictions (p01, p02, p03, p04, p05, p06, p07, p09, p10). Finally, 3DPHM analysis confirmed those 9 cases and brought 1 additional correct prediction (p08), resulting in correct prediction for all 10 cases.

### Secondary Outcomes: Optimization of Surgical Strategy Through 3D Modalities and Patient Outcomes

**Figure 4:**
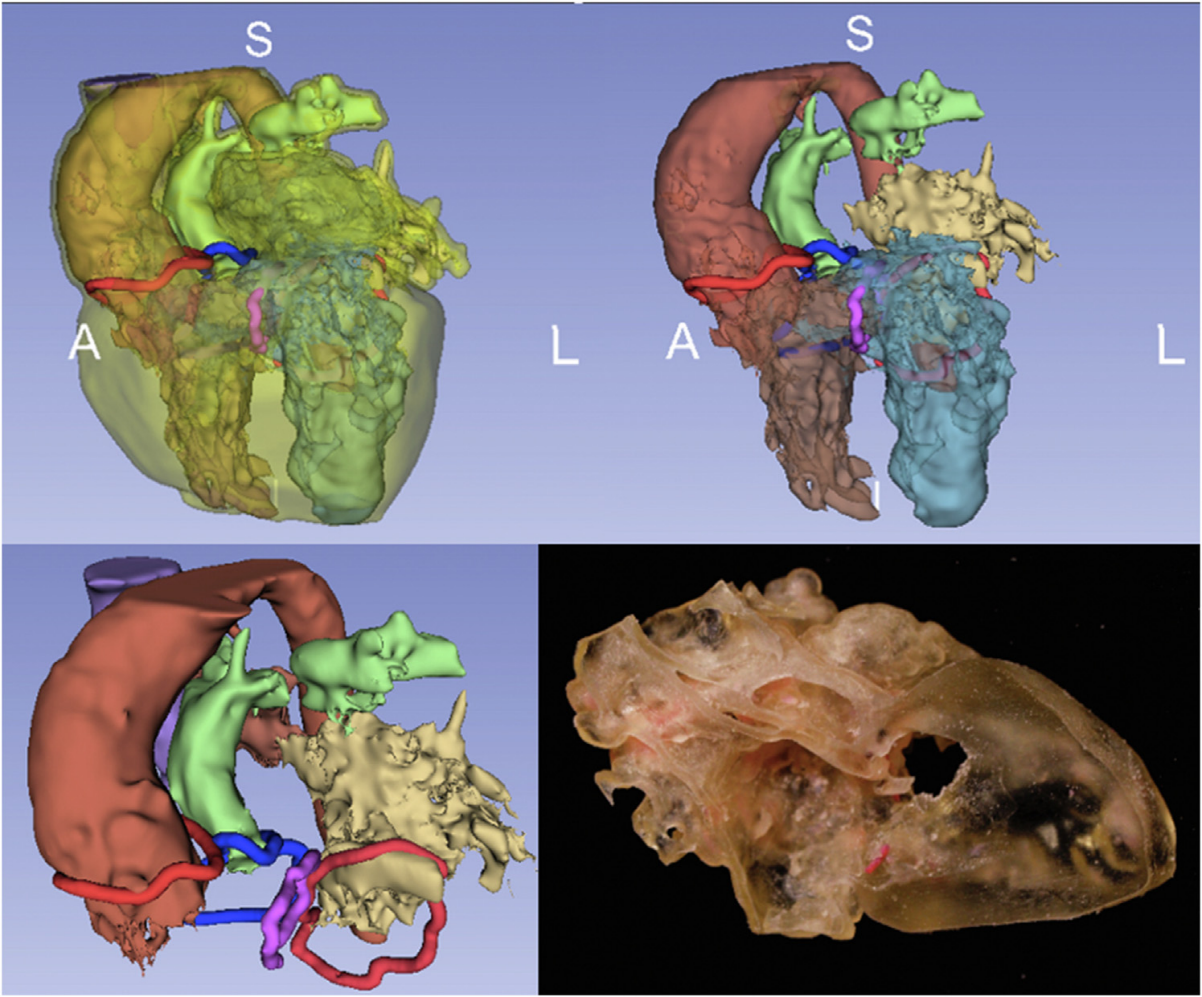
3D virtual valvular reconstruction and printed model of p04.

**Figure 5:**
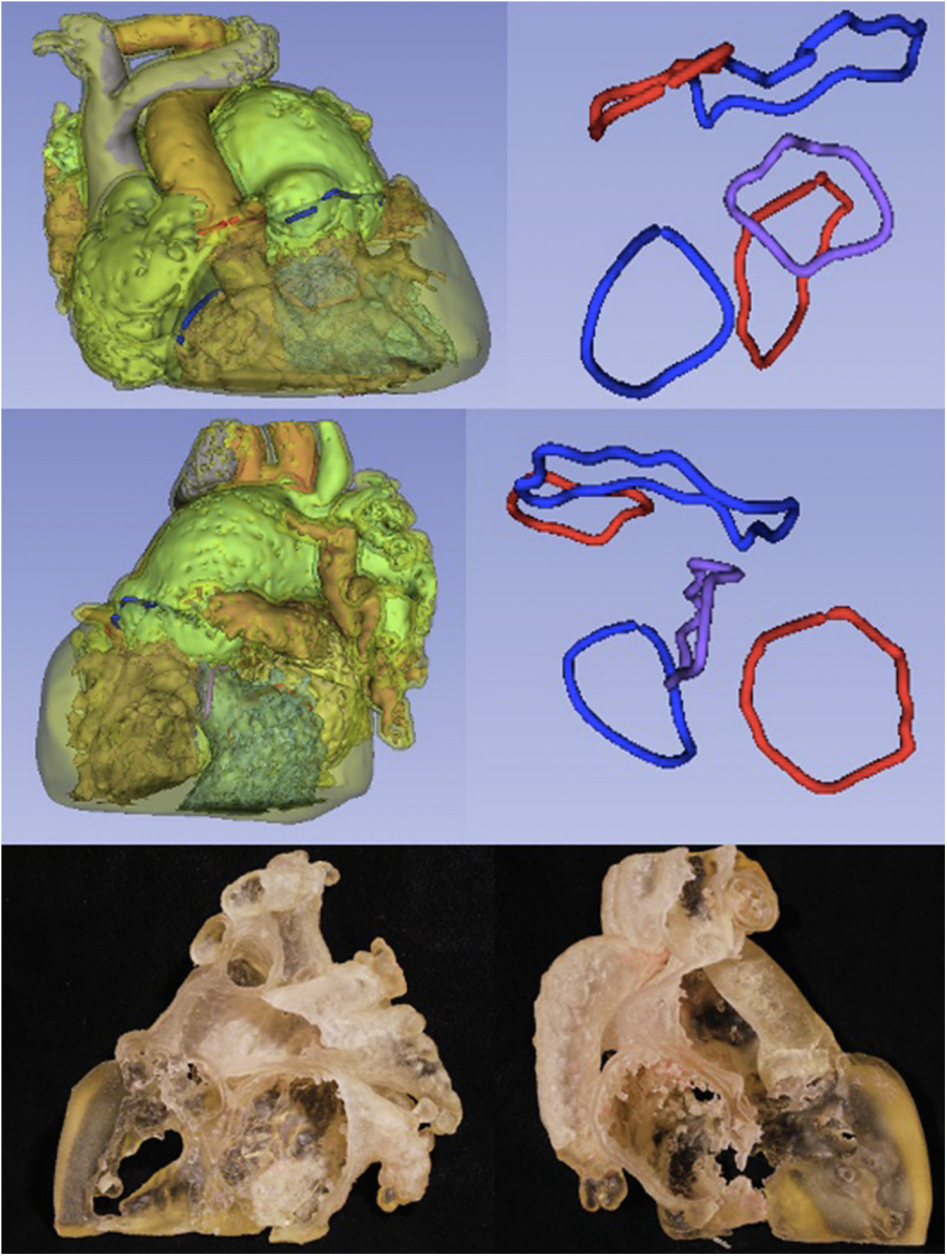
3D virtual valvular reconstruction and printed model of p10.

3D modalities contributed to optimization in surgical strategy for 5 cases. 3DVVR were involved in the modification of 4 cases (p01, p03, p04, p10), and 3DPHM in 1 case (p08). Concerning 3DVVR modifications, 2 Bex-Nikaidoh were preferred to outflow tract rotation (for p01 and p04) due to the better appreciation of pulmonary valve (PV) stenosis by the 3D rendering (Figure 4) (see p04 case description in Table E3). In addition, 2 outflow tract rotations were preferred to arterial switch operation as the result of better evaluation of PV stenosis for p03 and PV dilation for p10 due to the high risk of neoaortic valve insufficiency in case of arterial switch operation (Figure 5) (see p10 case description in Tables E3).

**Figure 6:**
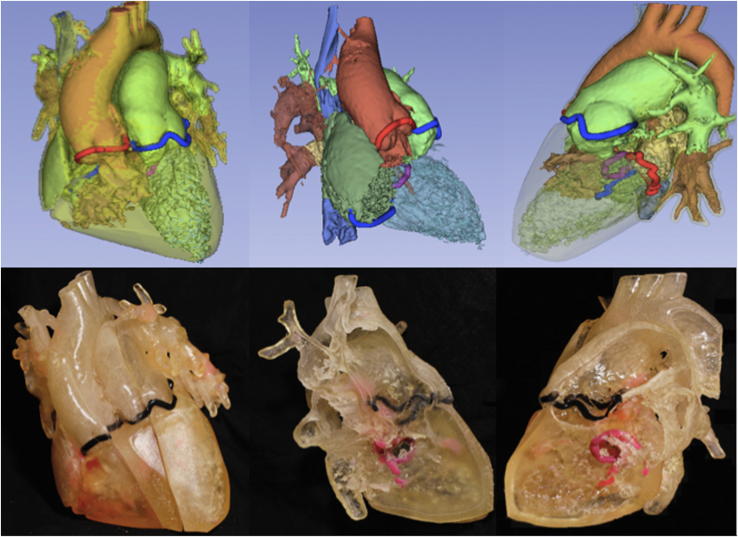
3D virtual valvular reconstruction and printed model of p08.

3DPHM contributed to correct the prediction for 1 patient (p08) (Figure 6). This case was a complex anatomical DORV with a side-by-side aortopulmonary position, a noncommitted ventricular septal defect (VSD) with inlet extension, and an important straddling of the tricuspid valve with a chordal attachment on the left side of the septum. This makes intraventricular repair (left ventricle [LV]-to-aorta baffle) impossible. Nikaidoh or outflow tract rotation was compromised as the result of complex abnormal coronary pathway. The left coronary artery gave rise to the interventricular artery, the circumflex artery, and the right coronary artery. An abnormal coronary artery with a suprasinusal ostium gave rise to conal and infundibular perforating arteries. This would have led to a high-risk root harvesting with potential coronary damage. Therefore, multimodal imaging advocated for single-ventricle palliation as the best surgical strategy. 3DVVR suggested a seemingly shorter distance between the LV and PA but was not sufficiently convincing for a biventricular repair with arterial switch operation. 3DPHM, with its 1:1 scale, gave the best appreciation of the actual short LV-to-PA distance for resectability of subpulmonary conus, making an arterial switch operation with LV-to-PA (neoaorta) baffling feasible. Retrospectively, the original heart team decision was a single-ventricle palliation, but peroperative findings changed the strategy to an arterial switch operation with LV to PA/neoartic valve baffling. In conclusion, 3DVVR was not convincing enough to support a biventricular repair. Only 3DPHM in this case allowed us to foresee intraoperative findings and to correctly anticipate the applied surgical strategy.

The mean intensive care unit length of stay was 9.4 days (±7 days). Hospitalization length of stay was 19.8 days (±11.6 days). In-hospital survival was 100%. P01 went through a single-ventricle palliation, which meant 3 operations at a 2-year interval. P06 was reoperated at 5 days for a revision of the intraventricular tunnelization due to residual left to right shunt. Three days after the reoperation, complete atrioventricular block motivated a bicameral permanent pacemaker implantation. P07 needed a reoperation for tricuspid repair due to complete rupture of a tricuspid chordae at 4 days postoperatively. Only 1 patient needed a permanent pacemaker after the reoperation. Survival could have been evaluated for 2 patients (p01 and p05), respectively 2 and 7 years after their operations.

## Discussion

The main result of our study showed that surgical strategy was correctly predicted in 50% (5/10) by multimodal imaging, 90% (9/10) by 3DVVR, and 100% by the 3DPHM. Both 3DVVR and 3DPHM improved the definitive surgical strategy prediction.

Our results are in accordance with current literature. Several studies demonstrate promising results concerning the use of 3D printing in preoperative planning. Valverde and colleagues^20^ reported a multicentric prospective case-crossover study including 10 centers, 80 pediatric cardiologists, and 22 surgeons using 3D models of various complex congenital heart disease, comparing the surgical indication using standard multimodality imaging alone with the same process with 3DPHM, finally confronted to surgical findings. In this study, 3D models were considered helpful in optimizing surgical planning in 19 of 40 patients (48%). No data were provided on specific anatomical structures or key elements triggering modification of the decision with a wide heterogeneity of included heart defects.

Ryan and colleagues^21^ retrospectively compared a presurgical 3DPHM group of 33 pediatric patients with DORV and dextro-TGA with a routine imaging group of 113 cases, showing a reduction trend of mean operative time for the 3DPHM group. These results mirror findings from Zhao and colleagues,^22^ who compared 8 DORV cases in the 3DPHM group with 17 cases in the control group. The 3DPHM group had shorter operative, cardiopulmonary bypass, aortic crossclamping, and mechanical ventilation times than the control group. These findings implicitly indicate 3DPHM could play a critical role in enhancing preoperative planning. However, Lau and Sun^27^ stressed that both studies did not achieve statistical significance, probably because of small sample size, rather than unfavorable outcomes.

Our study entails multiple strengths. First, the capacity of a multidisciplinary pediatric heart team to orchestrate and manage pre-, per-, and postoperative care of those complex cases allowed 3DPHM evaluation for complex cases planning. Second, we focused on DORV TGA-type exclusively, evaluating a homogenous group of patients. Third, the free and open-source 3D virtual reconstruction 3D Slicer software represents a low-cost and effective segmentation tool allowing easy deployment in clinical workflow.

Interestingly, 3DVVR was the most useful 3D modality for the optimization of surgical strategy. 3DVVR incremental value resided in the evaluation of relative size of valvular annuli and their relation to VSD. TTE offers noninvasive bedside valvulopathy assessment but lacks accuracy in measuring valvular dimensions in some patient categories compared with MRI.^28^^,^^29^ Nevertheless, MRI does not confer tridimensional intracardiac reconstruction. 3DVVR conveys a pragmatic solution to this. This modality could also alleviate the cost, time, and availability of 3DPHM. Nevertheless, 3DPHM was particularly helpful to evaluate 3DVVR findings at patients 1:1 scale, offering a realistic preoperative view and a better prediction of postoperative potential outflow tracts obstruction. Hence, the surgeon could consider with more confidence certain complex surgical strategies, such as outflow tract rotation. Other 3DPHM benefits, although not investigated in our study, are currently discussed in the literature^30^: clinical communication, discussion with the patient's family, and surgical rehearsal or training of complex cases. New 3D tools, such as virtual reality, are beginning to appear, with the potential of alleviating the need of printed models. Milano and colleagues^31^ reported a retrospective study of 10 patients with DORV with complex VSD types undergoing 3DPHM and virtual reality in the surgical planning. Multimodal imaging left 25% of patients with an univentricular repair, which was then reduced to 15% after 3DPHM evaluation, and only 5% after virtual reality. This latter option helped to consider biventricular repair, the arterial switch operation, for 95% of patients, in accordance with the actual surgical planning.

Lau and colleagues^32^ enrolled 29 practitioners to study the additional benefit of virtual reality and 3DPHM. The study demonstrated no significant differences between both technologies, but 72% of practitioners supported both the additional benefits of virtual reality and 3DPHM compared with multimodal imaging visualization. Whether virtual reality is able to challenge the need for 3DPHM remains to be investigated in larger clinical trials.

Several limitations of our study were identified. Primary and secondary end points could be inherently biased due to the nonmeasurability of human factors that drive decision-making. Important confounders such as the evolving experience of the surgeon, the awareness of alternative strategies, personal/institutional bias, preferences of referring provider, as well as risk aversion cannot be avoided. The retrospective virtual decision-making approach is not always correlated with real-time decisions secondary to potential clinical variables regarding a patient at the time of actual surgical planning. The same heart team took care of these cases several years ago, introducing a potential recognition bias in our study. We tried to minimize those biases with a multidisciplinary and consensual approach and a blinded analysis of the cases with an average surgery-to-study meantime of 19 months, but we acknowledge that they could still be present. Even if the choice of surgery strategy has inherently a subjective component, we believed that the present results are still valuable because they emphasized the objective part of our decision supported by imaging and that they could help other centers in their decision-making. Finally, we could not include all patients with DORV in our institution because of absence of CTA and MRI for the patients operated on the basis of echocardiographic imaging only.

### Clinical Studies and Future Evolution

3DVVR and 3DPHM demonstrated great potential to be routinely implemented for the surgical planning of patients with DORV. As the experience expands, the demand for reconstructions and models will increase and its use will be integrated systematically. 3D rendering of the heart represents strong clarifying tools to assess the complexity of intracardiac architecture, to identify critical steps, and to anticipate anatomical pitfalls, thus bringing strategic and technical solutions for surgical planning of patients with DORV. Future prospective studies are needed to quantitatively measure whether 3DPHM implementation reduces operative time, hospital length of stay, as well as morbidity and mortality rate. From there, cost–benefit analysis can be carried out to evaluate the efficiency of 3D modalities in the surgical planning of patients with DORV.

## Conclusions

Both 3DVVR and 3DPHM improved standard multimodal imaging for the surgical strategy planning of patients with complex DORV TGA-type according to our practice. 3D modalities contributed to strategy optimization in 5 of 10 cases. 3DVVR was involved in 4 optimizations and allowed us to better appreciate 3D relationships between great vessels, their valves, and VSD, which makes it the most useful 3D modality for surgical strategy planning. 3DPHM, contributing to optimize 1 case and correctly predicting all of them, was particularly helpful to assess 3DVVR findings at a 1:1 patient scale and improve the evaluation of any outflow tract obstruction, offering a realistic preoperative view. This retrospective study confirms the added value of 3DVVR and 3DPHM for surgical strategy planning and supports future prospective studies to assess their postoperative impact on patient outcomes.

### Conflict of Interest Statement

The authors reported no conflicts of interest.

The *Journal* policy requires editors and reviewers to disclose conflicts of interest and to decline handling or reviewing manuscripts for which they may have a conflict of interest. The editors and reviewers of this article have no conflicts of interest.

## Figures and Tables

**Table E1:** Patient characteristics at admission

Patients	Sex	Age, y	Weight, kg	Weight percentile	Height, cm	Height percentile	SpO_2_, %	Position of great arteries	Imaging to study interval, mo
p01	M	03	15.3	P50	96.3	P25	80	TGA	57
p02	F	02	9.28	P3-10	70	<P3	98	Side-by-side	14
p03	M	01	10.9	P25	88	P75	74	TGA	27
p04	M	06	18	P3-10	109	P3	65	TGA	34
p05	F	04	4.57	<P3	63	P3-10	75	TGA	42
p06	F	01	8.5	<P3	84.5	P25-50	96	Side-by-side	5
p07	M	02	12.5	P25-50	90	P50	75	TGA	4
p08	M	13	23.5	<P3	141	<P3	75	Side-by-side	3
p09	M	10	24.6	<P3	136	P10-P25	72	TGA	3
p10	F	2	9.5	<P3	84	P3-P10	86	TGA	2

*SpO*_*2*_, Oxygen saturation; *M*, male; *TGA*, transposition of the great arteries; *F*, female.

**Table E2:** Description of DORV types and associated findings

	DORV type	VSD	ASD	Valvular anomaly	Stenosis
p01	TGA	Subpulmonary with inlet extension	–	–	Subpulmonary and valvular
p02	Side-by-side	subaortic and trabecular	–	–	Subaortic
p03	TGA	inlet	Ostium primum (OP) + otium secundum (OS)	Mitral valve (MV), pulmonary valve (PV)	Subpulmonary
p04	TOF	Subaortic and trabecular	OS	–	Sub-, supra-, and pulmonary (hypoplasia of PT from RPA)
p05	TGA	Subpulmonary with posterior extension	OS	–	Subpulmonary and valvular
p06	TGA	Subpulmonary with posterior extension and trabecular	–	Straddling tricuspid valve (TV) and MV	Subpulmonary and valvular
p07	Side-by-side	Restrictive perimembraneous	–	–	subaortic (TV attaches to septal crest), outlet septum
p08	TGA	Subpulmonary with posterior extension and trabecular	–	–	subpulmonary, leveled (hypoplasia PT, from LPA)
p09	Side-by-side	Inlet and trabecular	–	TV straddling	RVOTO (conus + anterior pap.)
p10	TGA	Subpulmonary and inlet	–	–	Subpulmonary
p11	TGA	Subpulmonary	OS	–	Aortic hypoplasia, PAs dilation

Case description with 3DVVR and 3DPHM of 3 DORV cases: p01: DORV TGA-type with severe pulmonary stenosis and subpulmonary VSD. The panel opted for an OTR after multimodality imaging step. The 3DM led in favor of a Nikaidoh due to severe pulmonary stenosis convincingly assessed after 3DVR, and confirm after the 3DPHM. An SVP was realized at the time of intervention according to medicosurgical decision. The temporal surgical expertise may explain the discrepancy. p03: TGA anteroposterior aortopulmonary orientation, with subpulmonary stenosis and inlet VSD. The pulmonary integrity suggested an ASO or OTR at multimodality imaging step. However, after the 3DVR visualization, OTR seemed more suitable due to better perceived stenotic pulmonary valve. 3DPHM confirmed the suspicion that convinced the team for an OTR. At the time of intervention, a Nikaidoh procedure was performed due to temporal surgical expertise. p05: DORV D-TGA type, with subvalvular and pulmonary stenosis, and subpulmonary VSD with posterior extension. 3DVR and 3DPHM resulted in a Nikaidoh procedure compared with a OTR after MI. In the medical records, OTR has been surgically attempted but a residual transpulmonary gradient made PVR necessary, adopting a Nikaidoh procedure. The 3DVR and 3DPHM would have facilitated the early adoption for a Nikaidoh procedure and could have reduced the operative time. p09: DORV TGA type, side-by-side aortopulmonary position, with inlet and trabeculated VSD and TV straddling as well as a medioventricular obstacle due to double conus. After multimodality imaging, SVP seemed better suited. The 3DVR brought consideration for biventricular reparation through ASO. 3DPHM changed the surgical strategy for a biventricular repair with ASO through representations of the aortic and pulmonary annuli relative matching size, the inlet VSD relation with the PV and the resectability of the second conus making a baffle from VSD to PV conceivable. Retrospectively, the original heart team decision was an SVP but peroperative findings changed the strategy for an ASO with VSD to neoartic valve baffling. The 3D modalities could have convinced for a straight away ASO. p11: DORV TGA-type, subpulmonary stenosis, hypoplastic aortic arch, and dilation of pulmonary arteries. An ASO was first considered after multimodal imaging, considering an integer pulmonary valve with a moderate dilated caliber. Then, OTR was finally preferred to ASO after 3DVR and 3DPHM, due to better appreciation of dilated pulmonary annulus at increased risk of neoaortic valve insufficiency in the future following an ASO. In the operative records, an ASO was attempted due to temporal surgical expertise. *DORV*, Double-outlet right ventricle; *VSD*, ventricular septal defect; *ASD*, atrial septal defect: *TGA*, transposition of the great arteries; *TOF*, tetralogy of Fallot; *PT*, pulmonary trunk; *RPA*, right pulmonary artery; *LPA*, left pulmonary artery; *RVOTO*, right ventricular outflow tract obstruction; *PA*, pulmonary artery.

**Table E3:** Case description with 3DVVR and 3DPHM of 3 DORV cases

p01: DORV TGA-type with severe pulmonary stenosis and subpulmonary VSD. The panel opted for an OTR after multimodality imaging step. The 3DM led in favor of a Nikaidoh due to severe pulmonary stenosis convincingly assessed after 3DVR and confirm after the 3DPHM. SVP was realized at the time of intervention according to medicosurgical decision. The temporal surgical expertise may explain the discrepancy.
p03: D-TGA anteroposterior aortopulmonary orientation, with subpulmonary stenosis and inlet VSD. The pulmonary integrity suggested an ASO or OTR at multimodality imaging step. But after the 3DVR visualization, OTR seemed more suitable due to better perceived stenotic pulmonary valve. 3DPHM confirms the suspicion that convinced the team for an OTR. At the time of intervention, a Nikaidoh procedure was performed due to temporal surgical expertise.
p04: DORV D-TGA type, with subvalvular and pulmonary stenosis, and subpulmonary VSD with posterior extension. 3DVR and 3DPHM resulted in a Nikaidoh procedure compared to a OTR after MI. In the medical records, OTR has been surgically attempted but a residual transpulmonary gradient made PVR necessary, adopting a Nikaidoh procedure. The 3DVR and 3DPHM would have facilitated the early adoption for a Nikaidoh procedure and could have reduced the operative time.
p08: DORV TGA type, side-by-side aortopulmonary position, with inlet and trabeculated VSD and TV straddling as well as a medioventricular obstacle due to double conus. After multimodality imaging, SVP seemed to better suit. The 3DVR brought consideration for biventricular reparation through ASO. 3DPHM changed the surgical strategy for a biventricular repair with ASO through representations of the aortic and pulmonary annuli relative matching size, the inlet VSD relation with the PV and the resectability of the second conus making a baffle from VSD to PV conceivable. Retrospectively, the original heart team decision was an SVP but perioperative findings changed the strategy for an ASO with VSD to neoaortic valve baffling. The 3D modalities could have convinced for a straight away ASO.
p10: DORV TGA-type, subpulmonary stenosis, hypoplastic aortic arch and dilation of pulmonary arteries. An ASO was first considered after multimodal imaging, considering a integer pulmonary valve with a moderate dilated caliber. Then, OTR was finally preferred to ASO after 3DVR and 3DPHM, due to better appreciation of dilated pulmonary annulus at increased risk of neoaortic valve insufficiency in the future following an ASO. In the operative records, an ASO was attempted due to temporal surgical expertise.
